# Shell Extracts from the Marine Bivalve *Pecten maximus* Regulate the Synthesis of Extracellular Matrix in Primary Cultured Human Skin Fibroblasts

**DOI:** 10.1371/journal.pone.0099931

**Published:** 2014-06-20

**Authors:** Thomas Latire, Florence Legendre, Nicolas Bigot, Ludovic Carduner, Sabrina Kellouche, Mouloud Bouyoucef, Franck Carreiras, Frédéric Marin, Jean-Marc Lebel, Philippe Galéra, Antoine Serpentini

**Affiliations:** 1 UMR BOREA « Biologie des ORganismes et Ecosystèmes Aquatiques », MNHN, UPMC, UCBN, CNRS-7208, IRD-207, Université de Caen Basse-Normandie, IBFA, Campus 1, Science C, Caen cedex 5, France; 2 Laboratoire Microenvironnement cellulaire et pathologies (MILPAT), EA 4652, SFR 146 ICORE, Université de Caen Basse-Normandie, Faculté de Médecine, CHU niveau 3, Caen cedex 5, France; 3 Equipe de Recherche sur les Relations Matrice Extracellulaire Cellules (ERRMECe), EA 1391, Institut des Matériaux, Université de Cergy-Pontoise, Cergy-Pontoise cedex, France; 4 UMR 6282 CNRS “Biogéosciences”, Université de Bourgogne, Dijon, France; Aix-Marseille University, France

## Abstract

Mollusc shells are composed of more than 95% calcium carbonate and less than 5% of an organic matrix consisting mostly of proteins, glycoproteins and polysaccharides. Previous studies have elucidated the biological activities of the shell matrices from bivalve molluscs on skin, especially on the expression of the extracellular matrix components of fibroblasts. In this work, we have investigated the potential biological activities of shell matrix components extracted from the shell of the scallop *Pecten maximus* on human fibroblasts in primary culture. Firstly, we demonstrated that shell matrix components had different effects on general cellular activities. Secondly, we have shown that the shell matrix components stimulate the synthesis of type I and III collagens, as well as that of sulphated GAGs. The increased expression of type I collagen is likely mediated by the recruitment of *trans*activating factors (Sp1, Sp3 and human c-Krox) in the −112/−61 bp *COL1A1* promoter region. Finally, contrarily to what was obtained in previous works, we demonstrated that the scallop shell extracts have only a small effect on cell migration during *in vitro* wound tests and have no effect on cell proliferation. Thus, our research emphasizes the potential use of shell matrix of *Pecten maximus* for dermo-cosmetic applications.

## Introduction

Molluscan shells are known to be composed of CaCO_3_ crystals embedded in a thin organic cell-free matrix layer that is essential for controlling the shell biomineral deposition. This matrix contains several macromolecules, including polysaccharides (e.g., chitin), proteins and glycoproteins that are present both in inter- and intracrystalline locations [1]–[3].

Studies focused on the analysis of protein components of these shell organic matrices from marine molluscs have identified a large number of these proteins [3]–[10]. Among this wide variety of shell proteins, some of these molecules have structural similarities with proteins found in higher vertebrates, especially in humans [11]–[19]. The observed similarity between some of the mollusc shell proteins and human proteins suggested some functional analogies, justifying *a posteriori* the usefulness to test the biological effects of shell extracts on many mammalian tissues. For example, studies on nacre extracted from the pearl oyster *Pinctada maxima* demonstrated that this biomaterial is biocompatible and exhibits osteogenic activity [20]–[23]. Moreover, implanting nacre powder in animal skin results in enhanced fibroblast activity and synthesis of the dermal extracellular matrix [24]. Pereira Mouries and coll [25] suggested that the presence of signalling molecules and diffusible factors in molluscan shell extracts explain such effects on mammalian tissues, such as bone and skin. In an independent manner, it was shown that scallop shell extracts enhance the turnover rate of the epidermal layer and increase the efficiency of the recovery of UV-injured rat dorsal skin [26]. These properties of scallop shell extract suggest that it may be a suitable cosmetic material [27]–[30] in particular for wound healing and skin repair.

Wound healing is a complex physiological process involving an integrated response by many different cell types controlled by a variety of cytokines/growth factors. Generally, wound healing involves sequential and overlapping processes corresponding to denaturation and necrosis of wounded tissues, inflammation, granulation tissue formation, and tissue remodelling by the restoration of physiological structure and function [31]–[32]. During the initial inflammatory phase of wound healing, fibroblasts migrate to the wound, where they synthesise and later remodel new extracellular matrix material, of which collagen is the main component [33]. Fibroblasts represent the main cellular population of the dermis. Their major function is to maintain extracellular matrix (ECM) homeostasis [34]–[35]. In the physiological situation, there is a balance between synthesis and degradation of the matrix components, including proteoglycans and collagen.

To analyze the cellular processes, such as fibroblast proliferation and migration in response to the growth factors that are present in a wound, various *in vitro* cell culture systems have been used [36]–[37]. Such models simplify and standardise the system compared with the more complex *in vivo* situation. In addition, this approach is suitable to assess biomaterials for their potential, at least in a preliminary step, to promote wound repair by stimulating cell proliferation, ECM synthesis and for their biocompatibility.

The present study investigates the effect of *Pecten maximus* shell fractions obtained from two different extractions on human dermal fibroblasts *in vitro*. The data indicate that the scallop shell organic matrix contains molecules involved in extracellular matrix synthesis and transcription factor stimulation.

## Materials and Methods

### 2.1. Ethic Statement

Human dermal samples were obtained from skin biopsies of healthy donors undergoing mammary hypertrophy surgery. All patients signed an informed consent agreement form. This study with human sample from Dept. Surgery Service of St Martin Clinic (Caen, France) was approved by the local Ethics Committee for research with human samples (Comité de Protection des Personnes Nord Ouest III) of the “Centre Hospitalier Universitaire” of Caen.

Field sampling did not require specific permissions. No endangered or protected species were involved.

### 2.2. Shell Matrices Extractions

The collection of shells of the scallop *Pecten maximus* and their reduction into fine powder was performed by Copalis (Boulogne-Sur-Mer, France). Briefly, the shells were collected from various fisheries located along the channel coast of France. Shells were brushed and incubated in NaOCl (10%, v/v) overnight to remove superficial organic contaminants. The shell calcified layers (nacre and prisms) were then thoroughly rinsed with deionised water, dried and then crushed into fine powder (<200 µm).

All subsequent extractions were performed at 4°C. The acid extraction was prepared using a protocol employed by one of the co-authors, with some modifications [38]–[39]. Shell powder was decalcified overnight in cold dilute acetic acid (10%, v/v) that was progressively added (250 µl every 10 sec). The solution was centrifuged at 3250 g for 30 min at 4°C. The resulting pellet, corresponding to the acid-insoluble matrix (AIM), was rinsed several times with MilliQ water, freeze-dried and weighed. The supernatant, corresponding to the acid-soluble matrix (ASM), was extensively dialysed (3.5 kDa cut-off, Spectra/Por dialysis membrane) against 10 L of MilliQ water for 3 days (several water changes) before being freeze-dried and weighed.

Water-soluble matrix (WSM) was obtained by suspending shell powder in MilliQ water (100 g/L) for 3 days at 4°C with continuous stirring. The solution was centrifuged at 3200g for 30 min at 4°C. The supernatant was subsequently freeze-dried and the WSM pellet weighed. To be sure that calcium did not interfere with the WSM extract, a control using CaCO_3_ salt was included in the experiments. In parallel to the WSM extraction, a CaCO_3_ extract was performed using the same steps as the WSM extraction.

All extracts were resuspended in phosphate-buffer saline (4 mg/ml) and filtered (0.22 µm mesh) before use.

### 2.3. Cell Culture

Skin samples minced in small squares (1 cm^2^) were enzymatically digested for 15 h with thermolysin (40 U) supplemented with gentamycin (4 µg/ml) and Fungizone (2.5 µg/ml) to facilitate separation of the epidermis from the dermis. Fibroblasts were extracted from the dermis by an additional treatment with *Clostridium hystolyticum* type 1 collagenase (2 mg/ml) for 2 h at 37°C. The cell suspension was filtered through a 70-µm cell strainer and then centrifuged for 10 min at 300 g to obtain a cell pellet. The pellet was resuspended in Dulbecco’s modified Eagle’s medium (DMEM) supplemented with 10% foetal calf serum (FCS) and with antibiotics, antifungals (gentamycin, 4 µg/ml, Fungizone 2, 5, µg/ml, ciprofloxacin, 10 µg/ml) and 2% Mycokill. Cells were seeded at 2.5×10^6^ cells in 75 cm^2^ culture flasks in DMEM with 10% FCS and antibiotics in a 5% CO_2_ environment. They were passaged with a trypsin (0.05%) and EDTA (0.25 mM) solution after reaching confluence. All the experiments were performed on cells between passages 3 and 8.

### 2.4. WST-1 Assay

Cells were seeded onto 96-well microplates at a density of 2000 cells/well. After reaching 80% confluency, the cells were incubated in DMEM with 2% FCS in the absence or presence of shell matrix extracts or CaCO_3_ extract for 24 h, 48 h and 72 h. The medium was then removed and 100 µl of WST-1 reagent (WST-1 cell proliferation kit; dilution 1∶40 in DMEM) was added for 40 min. Absorbance was measured at 450 nm and 630 nm with a microplate reader.

### 2.5. Crystal Violet Assay

Cells were seeded onto 96-well microplates at a density of 2000 cells/well. After reaching 80% confluency, the cells were incubated in DMEM with 2% FCS in the absence or presence of shell matrix extracts or CaCO_3_ extract for 24 h, 48 h and 72 h. The medium was then removed, and the wells were washed twice with PBS. The cells were stained with 0.1% crystal violet dissolved in a PBS/Ca^2+^ solution for 30 min. The stained product was subsequently washed three times with PBS. Finally, the stained cells were solubilised in 20% acetic acid solution for 15 min. Absorbance was measured at 600 nm with a microplate reader.

### 2.6. Type I Collagen and MMP-1 ELISAs

At the end of the incubations, the cells were washed twice with PBS. Then, fibroblasts were lysed in RIPA buffer supplemented with leupeptin (1 µg/ml), PMSF (10 µg/ml), aprotinin (1 µg/ml), and pepstatin (1 µg/ml) as described previously [40]. Samples were centrifuged (12,000 g for 30 min at 4°C), and supernatants containing cellular proteins were stored at −20°C until analysis. Protein concentration was measured using a Protein Assay kit. Type I collagen measurements were evaluated in the culture media with the CICP MicroVue Bone Health kit, according to the manufacturer’s instructions. Absorbance was determined at 405 nm with a microplate reader. Active MMP-1 was assayed with the Fluorokine E Human active MMP-1 Fluorescent Assay following the manufacturer’s recommendations. Absorbance was measured at 405 nm with a microplate reader. The results were normalised to the cell layer protein amounts.

### 2.7. RNA Extraction and Real Time RT-PCR Analysis

Cells were seeded onto 12-well microplates and incubated in the absence or presence of shell matrix extracts for 48 h and 96 h without removal of the culture medium. Total RNA was extracted with TRIzol according to the manufacturer’s instructions. A total of 1.5 µg of total RNA was treated with 1.5 U DNase I at room temperature for 15 min to remove any DNA contaminants.

Reverse transcription was conducted using 1 µg of total RNA treated with DNase I, 20 µM oligodT, 200 U Moloney Murine Leukaemia Virus Reverse Transcriptase (MMLV-RT), 40 U RNaseOUT and 20 µM of each dNTPs. Real-time PCR was performed in an ABI Prism SDS 7000 thermocycler. All procedures were conducted in triplicate. Controls of non-template cDNA were included in the PCR experiments. The sequences of the forward and reverse primers were designed using Primer Express software (Table 1A). Amplifications were performed in 96-well plates for a total volume of 15 µl containing 5 µl of 1∶100 diluted cDNA samples obtained from reverse transcription, 7.5 µL of 2X SYBR Green Mastermix and both primers (200 nM final concentration for each primer). The amplification conditions were 40 cycles of 10 sec at 95°C and 60 sec at 60°C, followed by the protocol for the melting curve: 80 cycles of 10 sec with an increase of 0.5°C between each cycle from 55°C to 95°C. The melting curve was used to check whether the amplification products exhibited the expected Tm. The mRNA amount was normalised to *RPL13A* mRNA, and analysis of relative gene expression was performed by using the 2^−ΔΔCt^ method.

**Table 1:** Primers used in real time RT-PCR experiments (**A**) and oligonucleotides used in gel retardation assays (**B**).

A			
gene of interest	forward primer (orientation 5′–>3′)	reverse primer (orientation 5′–>3′)	amplicon
*RPL13A*	GAGGTATGCTGCCCCACAAA	GTGGGATGCCGTCAAACAC	75-pb
*COL1A1*	CACCAATCACCTGCGTACAGAA	CAGATCACGTCATCGCACAAC	118-pb
*COL1A2*	AAAACATCCCAGCCAAGAACTG	TCAAACTGGCTGCCAGCAT	91-pb
*COL3A1*	TCTTGGTCAGTCCTATGCGGATA	CATCGCAGAGAACGGATCCT	89-pb
*MMP-1*	GAAGCTGCTTACGAATTTGCCG	CCAAAGGAGCTGTAGATGTCCT	122-pb
*TIMP-1*	GTGTCTGCGGATACTTCCACAG	AGCTAAGCTCAGGCTGTTCCAG	131-pb
*p65*	TAGGAAAGGACTGCCGGGAT	CCGCTTCTTCACACACTGGA	101-bp
**B**			
name	Oligonucleotide sequence (orientation 5′–>3′)		
−112/−61 α1(I)	AGGCAGCTCTGATTGGCTGGGGCACGGGCGGCCGGCTCCCCCTCTCCGAGGG		
+2817/+2845 α1(II)	AGCGCAGCTGGCCCCGCCCCTGCGCCGGC		

### 2.8. Nuclear Extracts and Gel Retardation Assays

Nuclear extracts were prepared using the Andrews and Faller method [41]. After 48 h or 96 h incubation with shell matrices extract, fibroblasts were rinsed twice with cold PBS. Cell layers were scrapped in hypotonic buffer (10 mM Hepes, pH 7.9; 10 mM KCl; 1.5 mM MgCl_2_, 0.5 mM DTT, 0.5 mM phenylmethylsulfonyl fluoride, leupeptin, pepstatin A, and aprotinin at 10 µg/ml) and centrifuged for 3 min at 4,000 g. The supernatant was discarded, and the pellet was resuspended in hypertonic buffer (20 mM Hepes-KOH, pH 7.9; 25% glycerol (vol/vol); 420 mM NaCl; 1.5 mM MgCl_2_; 0.2 mM EDTA, 0.5 mM DTT, 0.5 mM phenylmethylsulfonyl fluoride, leupeptin, pepstatin A, and aprotinin at 10 µg/ml) and incubated on ice for 40 min. The sample was centrifuged (2 min, 12,000 g, 4°C), and the supernatant containing nuclear proteins was stored at −80°C until analysis.

Gel retardation assays were performed as previously described [42]. The probes used are shown in table 1B. The +2817/+2845 α1(II) probe contained potential binding sites for both Sp1 and Sp3 [43], and the −112/−61 α1(I) probe included potential Sp1 and human c-Krox binding sites [42]. Briefly, the probes were end-labelled with [γ-^32^P]dATP using T4 polynucleotide kinase. Nuclear extracts (7.5 µg) were incubated for 10 min at room temperature with the probes (10 fmol) in 20 µl of a specific-binding buffer and in a presence of 4 µg of poly(dI-dC). poly(dI-dC) used as a DNA nonspecific competitor. Samples were fractionated by electrophoresis for 2 h at 150 V on a 7.5% polyacrylamide gel in a 0.5X TBE (45 mM Tris-borate, 1 mM Na_2_EDTA) buffer and visualised by autoradiography.

### 2.9. Alcian Blue Staining

For alcian blue staining, cells were rinsed for 5 min with 0.1 N HCl (pH 1) or with 3% acetic acid (pH 2.5) to decrease the pH to reveal sulphated glycosaminoglycans or hyaluronic acid, a non-sulphated glycosaminoglycan, respectively [44]. Then, the cells were stained for 30 min with 1% 0.1 HCl or a 3% acetic acid alcian blue solutions (Alcian blue 8GX). Subsequently, the cells were washed 5 min twice with tap water and once with distilled water. The stained cells were photographed.

### 2.10. Cell Migration Assay

Kinetic analysis of the migration process was measured using time-lapse microscopy. Cells were seeded in 2 compartments separated by a silicon insert (Ibidi devices). At subconfluence, inserts were removed, cells were rinsed twice with PBS to remove non-adherent cells, and the culture medium was replaced by fresh medium containing or not ASM (500 µg/ml) or WSM (1000 µg/ml) or CaCO_3_ (1000 µg/ml) extract and supplemented with 0.1% SVF. Cell migration was followed using an inverted time-lapse microscope equipped with an environmental chamber at 37°C under 5% CO_2_. The microscope was controlled by Metamorph software. Images were taken every 30 min and recorded with a charged-coupled camera for 48 h. Cell migration was quantified using Image J software.

### 2.11. Immunofluorescence Analysis

Cells were grown on glass coverslips and incubated with shell extracts. The cells were then rinsed with PBS, fixed with 3% paraformaldehyde in PBS and washed with PBS containing 0.5% BSA. They were permeabilised with 0.1% Triton X100 in PBS and incubated for 1 h 30 min at room temperature with anti-vinculin or anti-β1 integrins antibodies. After washing, the coverslips were incubated with appropriate fluorescent secondary antibodies, Alexa Fluor 555-conjugated anti-mouse antibody or FITC-conjugated anti-mouse IgM antibody. Cell nuclei were stained using DAPI (4,6-diamidino-2-phenylindole dihydrochloride). Coverslips were mounted in Mowiol and examined with laser scanning confocal microscopy. For the negative controls, primary antibodies were replaced with PBS.

### 2.12. Data Analysis

The results are expressed as the means ± S.D. Each experiment was repeated at least three times, and the means were calculated from triplicates for each experiment. The significance of the differences between the mean values was estimated using Student’s *t*-test.

Densitometric analyses of the specific binding were performed with the ImageJ software and are presented in comparison to their respective control. The values are the mean of triplicate samples ±SD.

## Results

### 3.1. Shell Extracts Modulate Fibroblasts Metabolic Activity

**Figure 1:**
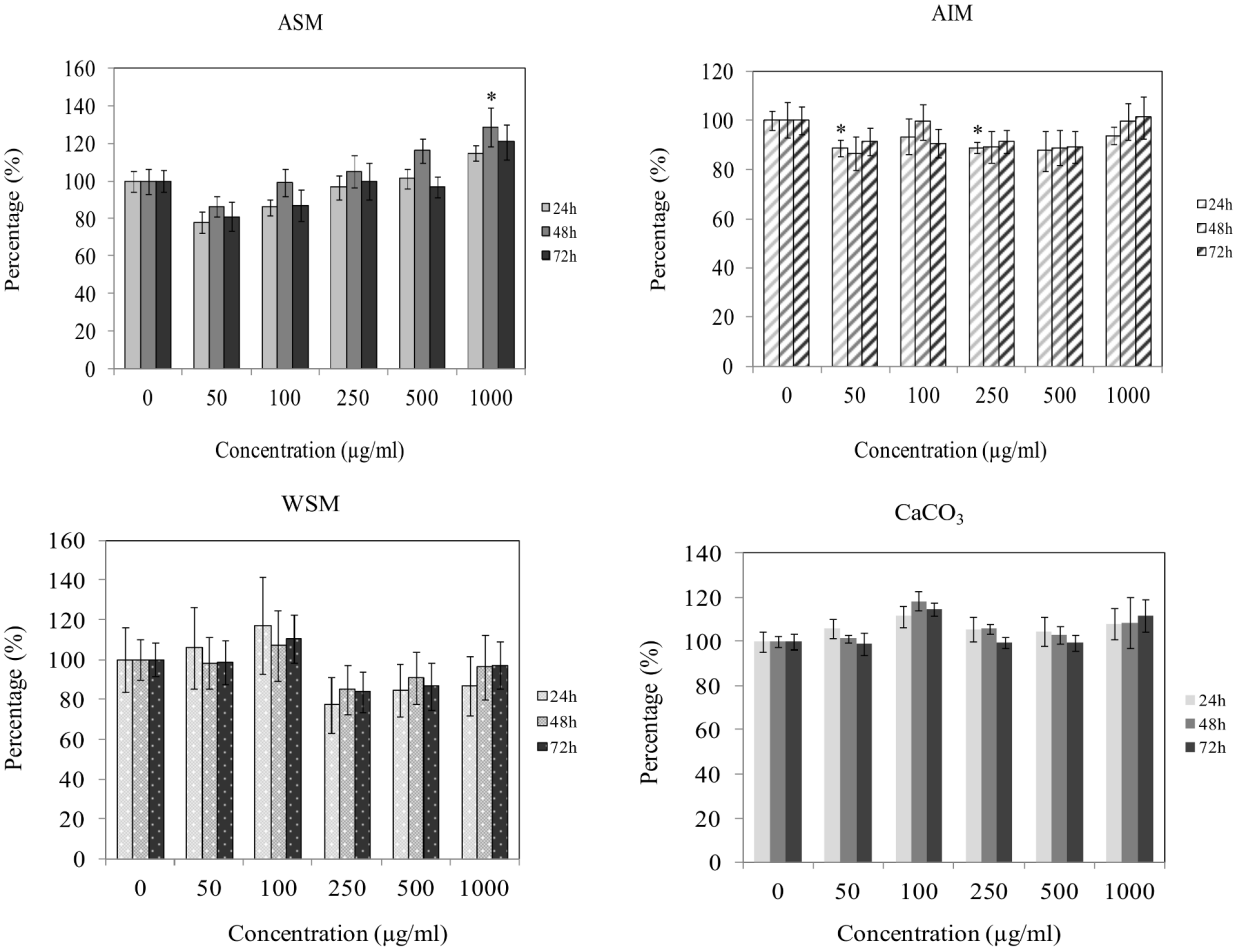
Effect of shell matrix extracts on fibroblast metabolic activity evaluated by WST-1 assay after culturing the cells in the presence of varying concentrations of extracts (50–1000 µg/ml) for 24 h, 48 h and 72 h. Statistical differences compared to controls are indicated by asterisks (*P<0.05), n = 4. ASM: acid soluble matrix, AIM: acid insoluble matrix, WSM: water soluble matrix, CaCO_3_: calcium carbonate used as control for WSM experiments.

The metabolic activity of fibroblasts exposed to different shell extracts was assayed using an WST-1 assay. In these experiments, the cells were exposed for 24, 48 or 72 h to various concentrations of extracts ranging from 50 to 1000 µg/ml (Fig. 1). No significant variation in the metabolic activity of cells was observed up to 500 µg/ml of ASM. However, a significant increase (p<0.05) was detected when cells were incubated for 48 h in the presence of 1000 µg/ml of ASM. For AIM, a significant decrease (p<0.05) appeared when cells were incubated for 24 h with 50 and 250 µg/ml. However, no significant variations were observed when cells were incubated for 48 and 72 h. Concerning the effects of WSM, because of higher standard deviation, the variations recorded did not indicate clear tendencies, although incubations performed with 250 to 1000 µg/ml exhibited a lower activity. However, these differences were not significant. For the CaCO_3_ extract, we did not record any difference with the blank test, regardless the concentrations and the incubation times.

### 3.2. Shell Extracts do not Modulate Cell Proliferation

**Figure 2:**
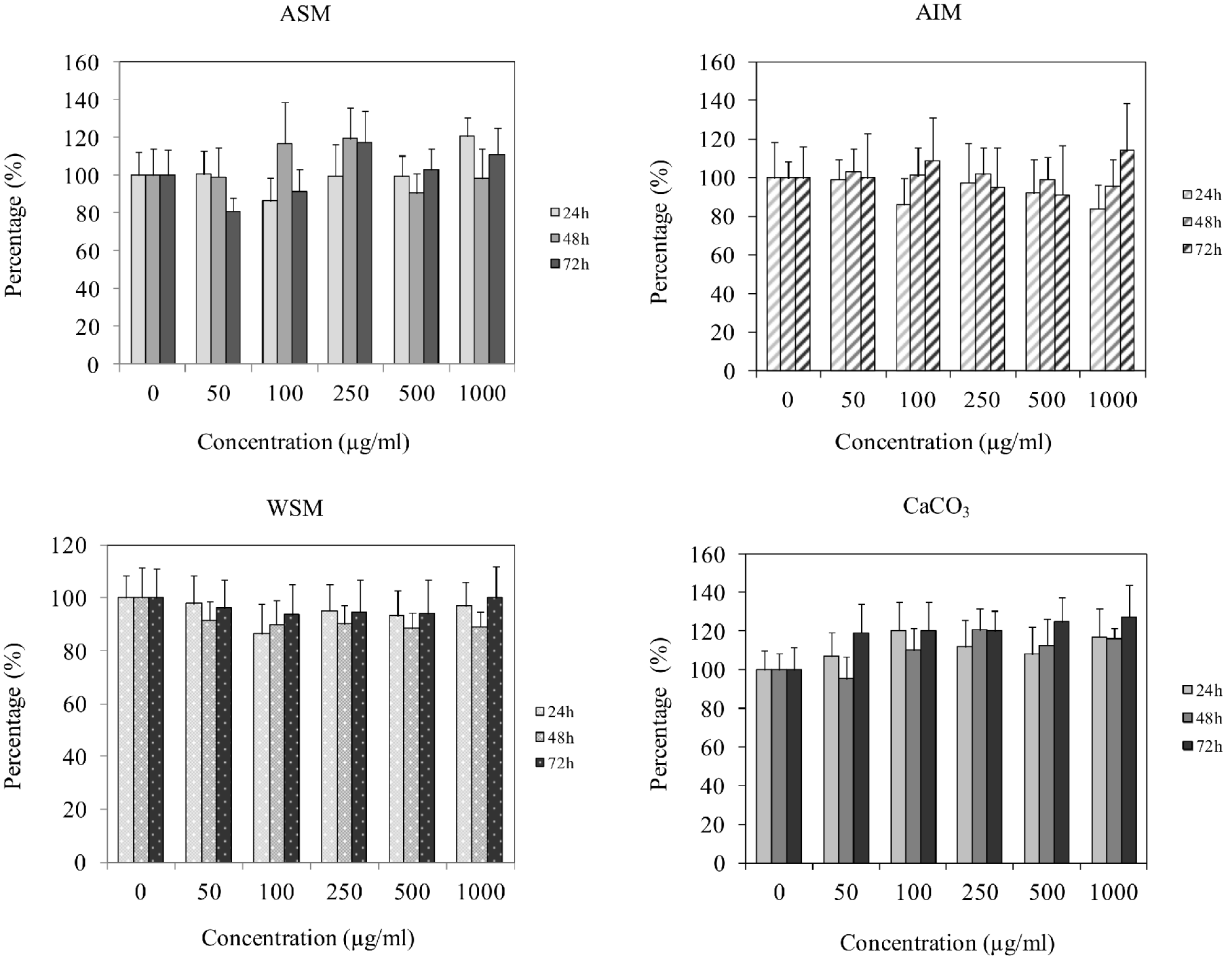
Effect of shell matrix extracts on fibroblast proliferation measured by crystal violet staining assay after culturing the cells in the presence of varying concentrations of extracts (50–1000 µg/ml) for 24 h, 48 h and 72 h. Statistical differences compared to controls were calculated and were not significant, n = 4. ASM: acid soluble matrix, AIM: acid insoluble matrix, WSM: water soluble matrix, CaCO_3_: calcium carbonate used as control for WSM experiments.

The number of fibroblasts exposed to different shell extracts was assayed using a crystal violet assay. In these experiments, cells were exposed for 24, 48 or 72 h to various concentrations of extract ranging from 50 to 1000 µg/ml (Fig. 2). The cell density was not significantly modulated compared with the control regardless of the shell extracts used.

### 3.3. Shell Extract Modulate ECM Gene Expression

**Figure 3:**
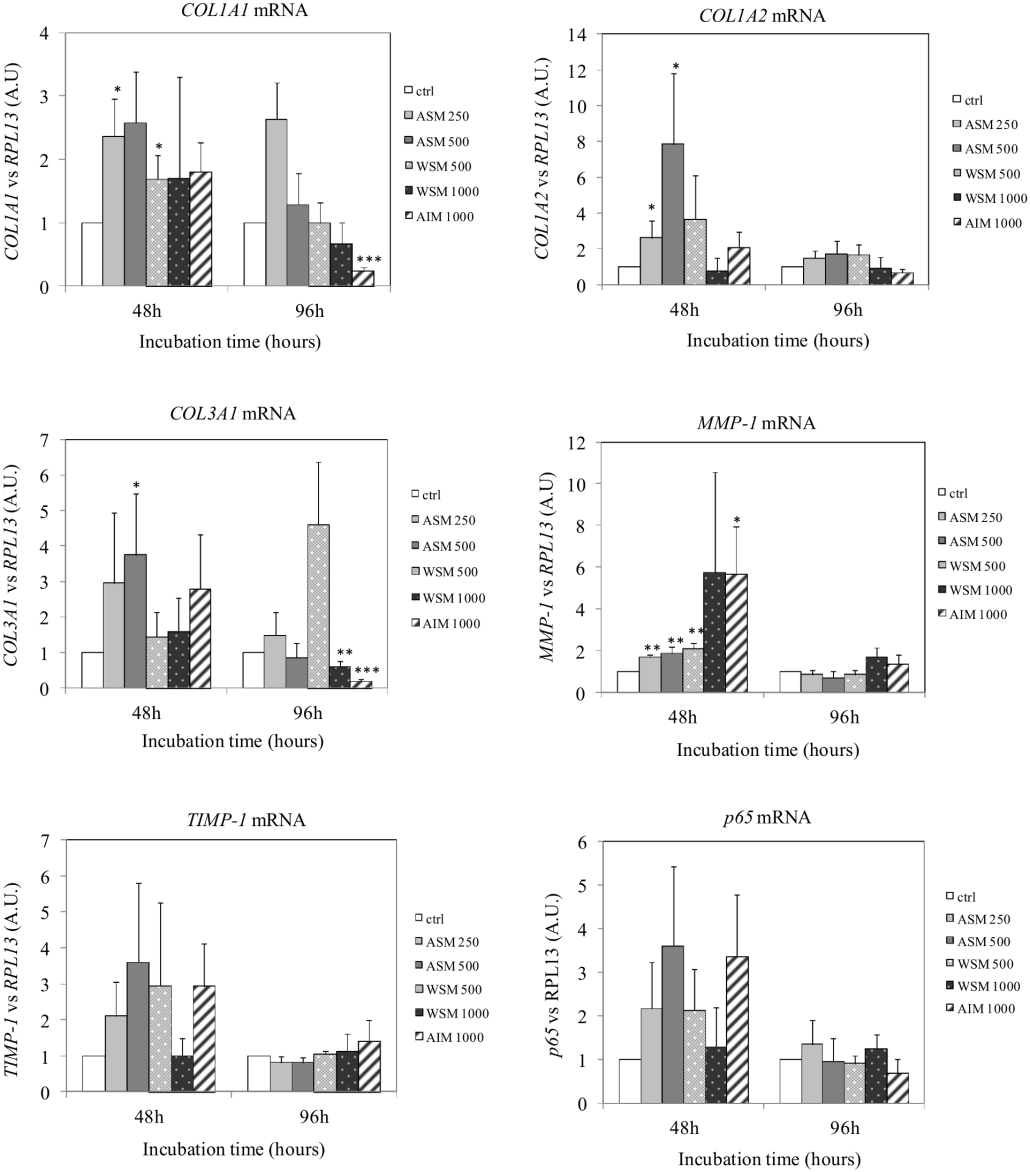
Effect of shell matrix extracts on *COL1A1, COL1A2, COL3A1, MMP-1 and TIMP-1* mRNA expression in fibroblasts. Total RNAs were reverse-transcribed into cDNA and real-time PCR assays were performed to determine *COL1A1*, *COL1A2*, *COL3A1*, *MMP-1* and *TIMP-1* mRNA expression levels. Values are the mean of triplicate samples ±SD. Statistical analyses were performed with the Student’s t-test (*P<0.05), n = 4.

We then studied the effects of shell extracts on the mRNA steady-state levels of some extracellular matrix components. On Fig. 3, we observed that ASM increased *COL1A1* (p<0.05), *COL1A2* (p<0.05), *COL3A1* (p<0.05) and *MMP1* (p<0.001) mRNA steady state levels (Fig. 3). Moreover, ASM slightly increased *TIMP1* and *p65* mRNA levels, but the effect was not significant. In contrast, AIM (1000 µg/ml) significantly decreased the levels of *COL1A1* (p<0.01) and *COL3A1* (p<0.001) expression level. In parallel, the level of *MMP-1* mRNA expression was significantly (p<0.05) increased when cells were incubated for 48 h with AIM. Moreover, AIM not significantly increased *TIMP-1* and *p65* mRNA amounts. WSM (500 µg/ml) enhanced *COL1A1* (p<0.05) and *MMP-1* (p<0.01) expression level significantly. Finally, we observed that WSM could also weakly but not significantly increase *COL3A1*, *TIMP-1* and *p65* mRNA amounts.

### 3.4. Shell Extracts Stimulate Extracellular Matrix Synthesis

**Figure 4:**
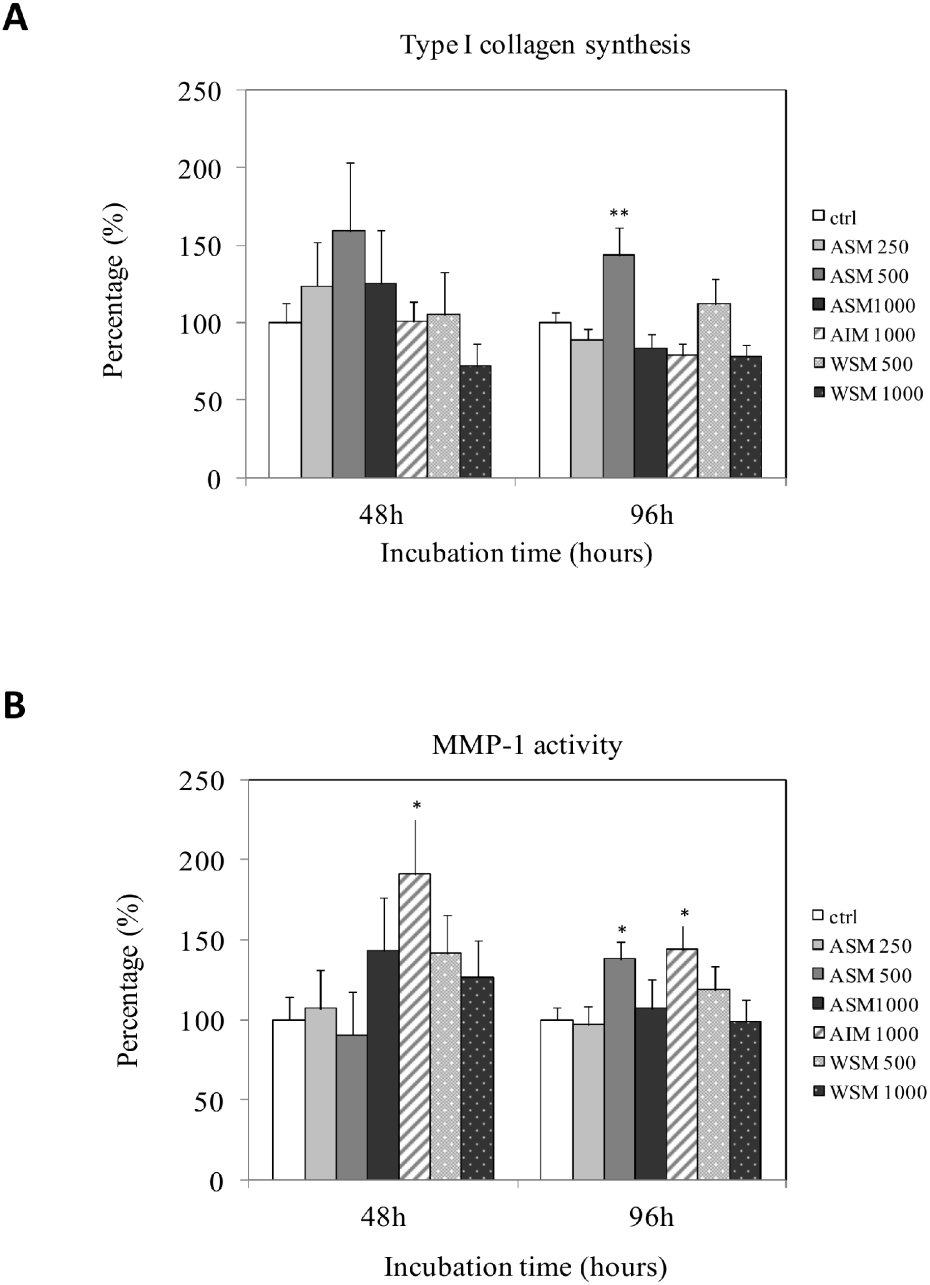
Effect of shell matrix extracts on fibroblast type I collagen synthesis and MMP-1 activity determined by ELISA assays after culturing the cells in the presence of varying concentrations of extracts (50–1000 µg/ml) for 48 h and 96 h. Statistical differences compared to controls are indicated by asterisks (*P<0.05, **P<0.01) n = 4. ASM: acid soluble matrix, AIM: acid insoluble matrix, WSM: water soluble matrix. The numbers next to ASM, AIM or WSM indicated the concentration (in **µ**g/ml) used in these experiments.

**Figure 5:**
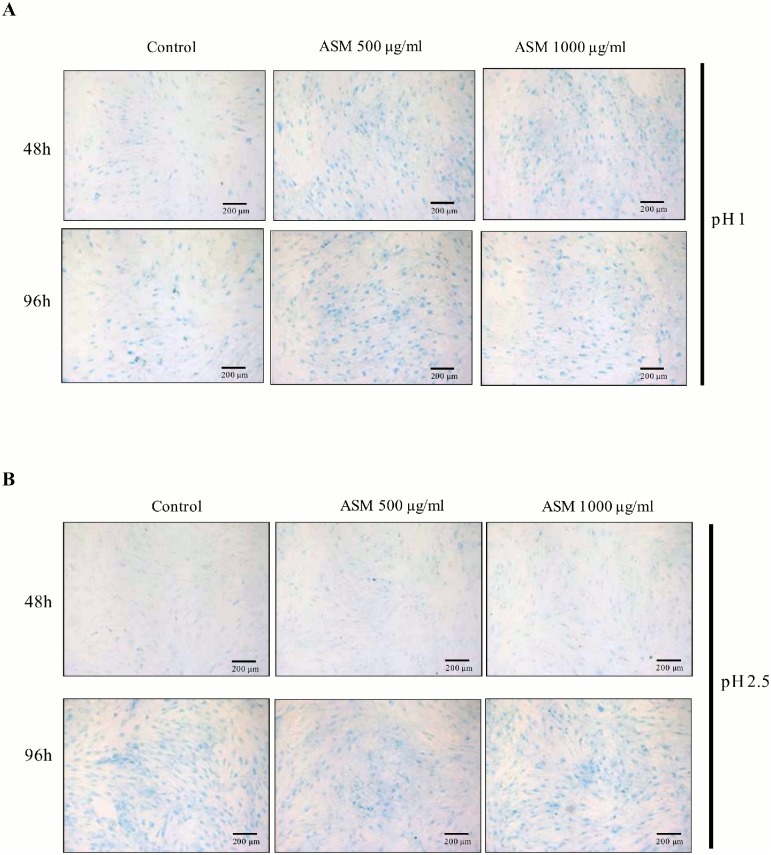
Effect of shell matrix extracts on fibroblast glycosaminoglycans (GAGs) synthesis evaluated by alcian blue staining assay after culturing the cells in the presence of varying concentrations of ASM (50–1000 µg/ml) for 48 h and 96 h. (A) Staining of sulphated glycosaminoglycans. (B) Staining of non-sulphated glycosaminoglycans. Results show one representative experiment, n = 3.

The effect of shell extracts on type I collagen synthesis and MMP-1 activity were evaluated using ELISA assays (Fig. 4). Type I collagen is one of the major constituents of the dermis extracellular matrix, and MMP-1 is the main enzyme responsible for its degradation. Moreover, GAG neosynthesis was also evaluated by the alcian blue staining method (Fig. 5).

ASM was demonstrated to increase type I collagen (p<0.01) synthesis and MMP-1 (p<0.05) activity. We found that ASM (500 µg/ml) stimulated collagen type I synthesis when the cells were exposed for 48 h. However, this stimulation was not significant. When the cells were incubated for 96 h with ASM (500 µg/ml), a significant (p<0.01) increase of the collagen type I synthesis was observed. AIM and WSM extracts did not modify fibroblasts collagen synthesis after 48 h or 96 h of exposure. ASM (500 µg/ml) significantly stimulated (p<0.05) MMP-1 activity when fibroblasts were exposed for 96 h. Moreover, AIM (1000 µg/ml) significantly enhanced (p<0.05) MMP-1 activity regardless of the incubation time. In contrast, no significant differences compared to the control were observed when the cells were incubated with WSM.

In addition, ASM (500 and 1000 µg/ml) specifically stimulated sulphated glycosaminoglycans synthesis (Fig. 5A) as indicated by alcian blue positive stain with pH 1 solution. On the contrary, ASM did not stimulate non-sulphated glycosaminoglycans synthesis (alcian blue solution, pH 2.5) when the cells were exposed for 48 h or 96 h (Fig. 5B).

### 3.5. Shell Extracts Stimulate Type I Collagen Gene Expression by Increasing the DNA-binding Activity of its Transactivators

**Figure 6:**
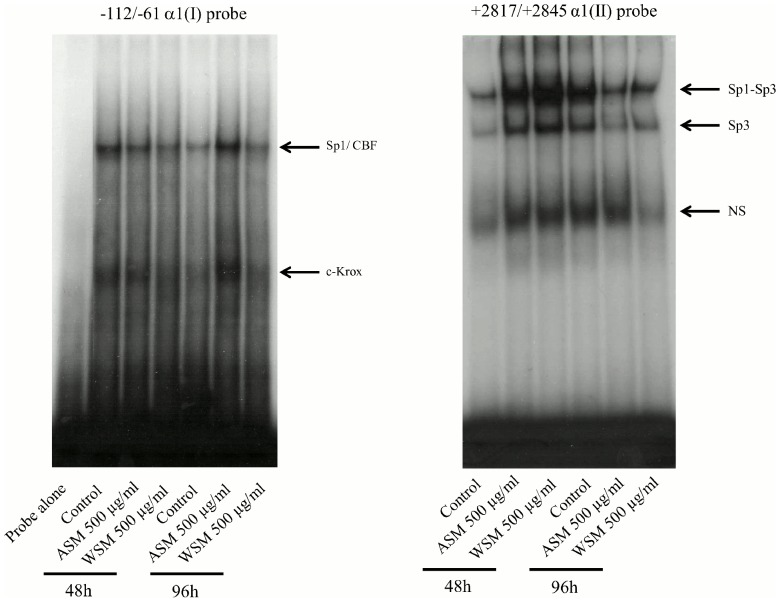
Effects of shell matrix extracts on the DNA binding activity of Sp1, Sp3 and c-Krox in fibroblasts. DNA binding activity of nuclear extracts from fibroblasts incubated with ASM or WSM was analyzed by gel retardation assay. Arrows indicate the complexes formed between DNA probes and nuclear proteins. NS: non-specific binding. In both panels, fibroblasts were incubated for 48****h in absence (line 1) or presence of 500 µg/ml of ASM (line 2) or WSM (line 3). In each panel fibroblasts were incubated for 96****h in absence (line 4) or presence of 500 µg/ml of ASM (line 5) or WSM (line 6). ø: probe alone. Results show one representative experiment, n = 3.

As ASM was demonstrated to increase *COL1A1* mRNA levels, we studied the DNA-binding activity of Sp1, Sp3, CBF and c-Krox, four well-known *COL1A1 trans*activators. DNA mobility shift assays using radiolabeled probes containing *cis*-acting elements of the human α1(I) collagen promoter and the human α1(II) collagen enhancer were performed (Fig. 6). ASM (500 µg/ml) likely increased the DNA binding of c-Krox, Sp1 and/or CBF when fibroblasts were incubated for 96 h (Fig. 6, left panel). Moreover, the gel retardation assay indicated that WSM (500 µg/ml) enhanced the DNA-binding activity of Sp1/Sp3 when fibroblasts were incubated for only 48 h (Fig. 6, right panel). Thus, ASM and WSM enhance *COL1A1* mRNA steady-state amounts likely through a transcription control involving c-Krox, probably CBF, Sp1 and Sp3, CBF and the three zinc-fingers being *trans*activators of *COL1A1*.

### 3.6. ASM and WSM Extracts Do Not Enhance Cell Migration, Neither Affect ECM Distribution

**Figure 7:**
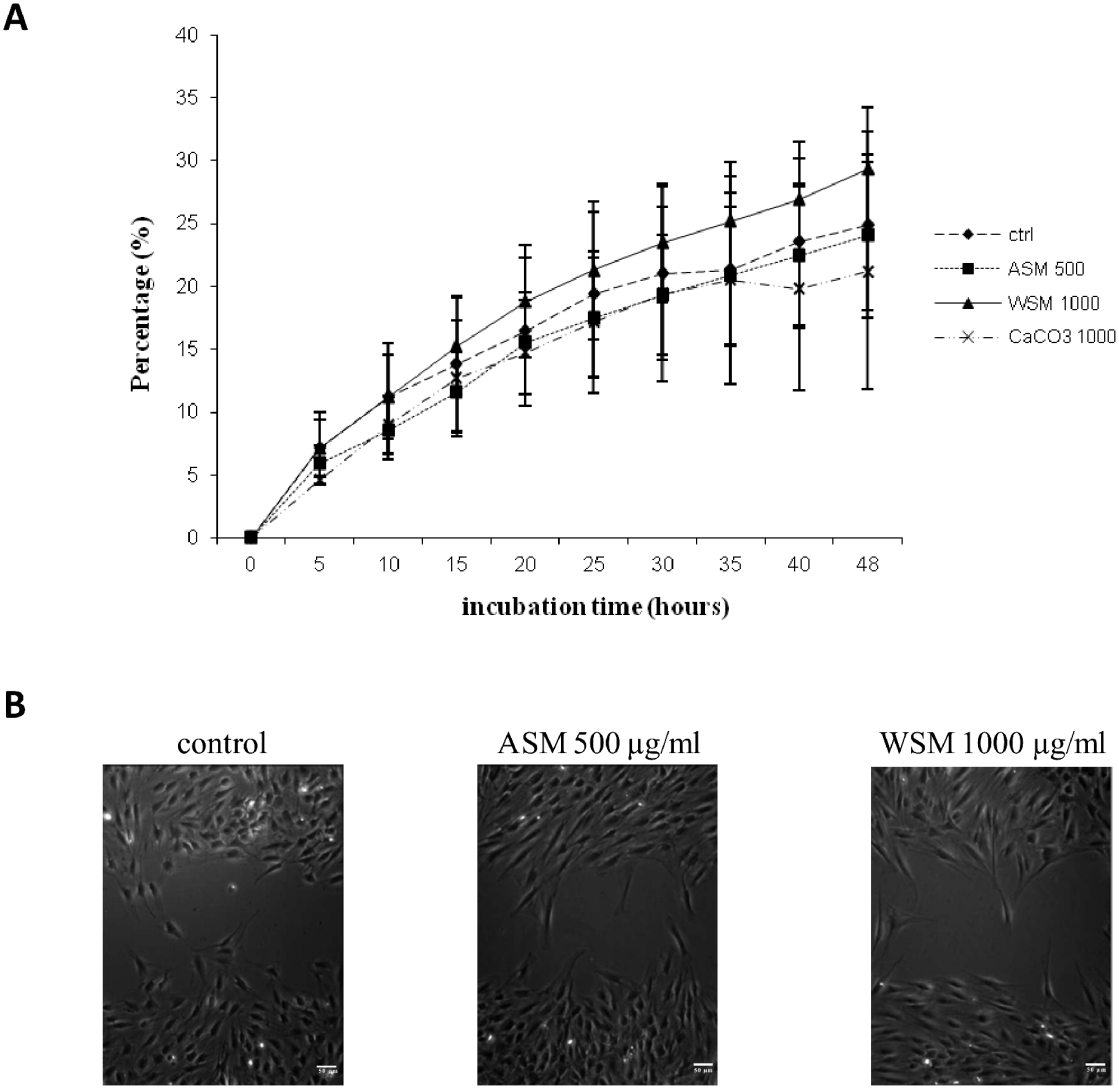
Effects of shell matrix extracts on fibroblasts migration after wound healing. (A) Percentage of filling of the *in* **** *vitro* wounds by fibroblasts in the presence or not of *Pecten maximus* shell extracts (ASM 500 µg/ml) and WSM 1000 µg/ml), or in the presence of CaCO_3_ (1000 µg/ml), during 48 h of incubation. Statistical differences compared to controls were calculated, n = 5. (B) Evolution of the filling of the *in vitro* wound after 48 h of incubation time, in the absence or presence of shell extracts (ASM 500 µg/ml and WSM 1000 µg/ml). Scale bars: 50 µm.

Fibroblast migration capacity in the presence or absence of ASM (500 µg/ml) and WSM (1000 µg/ml) was investigated using an *in vitro* wound closure assay (Fig. 7). After 48 h of incubation, the wound was not completely filled in control samples (24.9±7%). The addition of ASM in the culture medium did not influence the wound closure and therefore had no significant effect on the migratory capacity of fibroblasts in culture. However, a slight increase, approximately 4.5% of the wound closure, was observed in the presence of WSM (29.3±5% vs. 24.9±7%) after 48 h of incubation, but this increase was not significant.

Thus, in our experimental conditions, shell extracts of *Pecten maximus* did not appear to modulate the migration of fibroblasts in culture (Fig. 7B).

**Figure 8:**
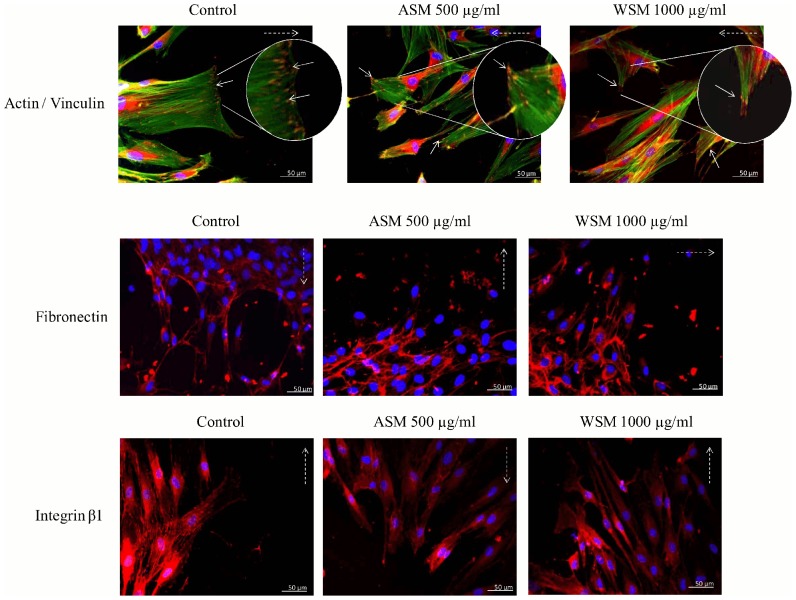
Effects of shell extracts on the location and distribution of fibronectine and β1 integrins receptors and cell elements involved in the cell migration, during *in vitro* wound healing. (A) Immunostaining of actin (green) and vinculin (red, indicated by arrows), (B) Immunostaining of fibronectin (red). (C) Immunostaining of integrin β1 (red). For all pictures, cell nuclei were staining with DAPI (blue). Dashed arrows represent cells migration direction. Results show one representative experiment, n = 4. Scale bars: 50 µm.

To assess whether shell extracts have an effect on ECM and the cell elements involved in cell migration, immunofluorescence experiments were performed (Fig. 8). The immunostaining of actin and vinculin revealed the presence of stress fibres, and focal contact structures were observed at the leading edges of spreading cells, illustrating the interaction between migrating cells and the culture support (Figure 8A). Fibronectin was stained and visualised in a fibrillar network, and this network appears to be denser under ASM and WSM conditions (Figure 8B).

The fibronectin receptors β1 integrins were also revealed to be randomly distributed all over the cell surface and also in a punctuated structure in association with focal contact or fibrillar adhesion (Figure 8C). Cell treatments with ASM and WSM extracts during cell migration after an *in vitro* wound assay did not reveal significant differences compared to the control samples. Even if the fibrillar network of fibronectin appears slightly different for migrating fibroblasts in the presence of ASM and WSM extracts, the global distribution of the different markers is typical of migrating cells.

## Discussion

The biomineralisation process is a widespread process in the animal kingdom. It allows the precipitation of ions, leading ultimately to the formation of mineral structures. Molluscan biomineralisation is characterized to a wide variety of structures and shell architectures, controlled by a complex organic matrix. A hypothesis has emerged that there is "an ancient heritage" shared by many calcifying metazoans leads to conservation of biological activities of these molluscan proteins on mammalian cells [2]–[45]. As an example, studies have demonstrated the bioactivity of nacre in the process of bone repair in vertebrates [21]–[23]. On the other hand, more recent studies have demonstrated that this biomaterial has biological activities in other “non-mineralized” tissue systems, and more particularly the cutaneous system. These results strongly suggest that the shell molecules possess the ability to modulate the synthesis of matrix components of the dermis in vertebrates [24]–[30].

Our main scope is the potential biological activities of the shell matrix compounds extracted from the organic fraction of the scallop *Pecten maximus* on the metabolism of human dermal fibroblasts in primary culture. We particularly focused on the expression of extracellular matrix elements involved in the anabolic pathway (for example, type I collagen) and also the expression of factors involved in the catabolic pathway (MMP-1). Moreover, the effects of shell extracts on cell migration and ECM localisation were investigated.

The results revealed that the ASM of *Pecten maximus* induced an increase (25%) of the metabolic activity of cells in the presence of 1000 µg/ml of extract. This effect was not accompanied by an increase in cell density, which suggests that ASM of *P. maximus* stimulates cell metabolism but not proliferation. Moreover, the AIM and the WSM conditions produced no effects on the metabolic activity or on the cell density. In the literature, studies have already demonstrated that such components affect the metabolism or proliferation of vertebrate cells maintained *in vitro* [25], [30], [46]–[48]. In particular, experiments performed on human dermal fibroblasts demonstrated that the ASM from the shell of the Japanese scallop *Patinopecten yessoensis* induces an increase in the metabolic activity of these cells after incubation in the presence of extract [30]. The results obtained in our work with extracts from the shell of *Pecten maximus* clearly indicate that these matrix components have biological activities, at least in regard to the general cellular activities in human dermal fibroblasts and are therefore in agreement with the literature data.

Expression of collagens such as type I collagen has been investigated after incubations in the presence of shell extracts. Collagens are major components of the ECM, and type I collagen is the most abundant protein found in the dermis. Other molecules, such as GAGs, appear as important elements that constitute the ECM. It was therefore important to observe the effect of shell extracts on the synthesis of GAGs. We have also analysed the effects of these extracts on the activity of MMPs (i.e., MMP-1), enzymes implicated in the ECM catabolic pathway (collagen degradation) and the expression of TIMPs, their inhibitors. Finally, from a mechanistic point of view, it was necessary to evaluate the effect of the shell components on the binding activity of transcription factors known to regulate the fibroblast synthesis of type I collagen.

The results indicated that ASM and the WSM increase the steady-state amounts of *COL1A1* and *COL1A2* mRNA, which encode the α1 and α2 type I collagen chains, respectively. These genes are generally expressed in a coordinated manner [49]. At the protein level, our results revealed an enhancement of the synthesis of type I collagen by fibroblasts when cells are exposed to shell extracts, especially in the presence of ASM. Moreover, the *COL3A1* mRNA level is also increased in the presence of ASM. Type III collagen is much more prevalent in young skin than in adult skin, and it plays an important role in the dermis. This collagen is particularly involved during tissue repair and wound healing [50], [51]. Thus, the fact that the molecules of the shell extracts from *Pecten maximus* can increase type I and type III collagen expression is particularly interesting in the context of potential applications in wound repair and anti-aging strategies. On the other hand, the results revealed an increase in sulphated GAGs in the presence of ASM. We suggest that these GAGs could be dermatan sulphate and/or chondroitin sulphate, which are the most sulphated GAGs located in the dermis. Furthermore, they are associated with proteins and form proteoglycans such as decorin or versican, which are PGs interacting with collagen type I, to establish a molecular network allowing the ECM assembly [52]. Our observations are consistent with several studies performed in rats and pig in the presence of shellfish extracts [26], [29], [30], [48]. Pearl extracts from the pearl oyster *Pteria martensii* placed on an injury on pig skin causes an increase in the granulation tissue, a removal of necrotic tissue, and an enhancement in the synthesis of type I collagen by fibroblasts at the repair site [48]. Moreover, this shell extract increased the expression at the protein and transcriptional level of collagen type I by a fibroblast cell line (NIH-3T3) *in vitro* [48]. All these results are in agreement with those obtained in our study and clearly suggest that shell extracts from *Pecten maximus* may have a possible involvement in tissue repair or in processes of tissue regeneration of both the dermis and epidermis.

What can be the molecular mechanism activated by scallop shell extracts? Our study gives the first elements of an answer, by testing which the molecules contained in the WSM and ASM could induce the transcription of *COL1A1* and enhance collagen synthesis. To this end, we evaluated the binding activities of transcription factors (c-Krox, CBF, Sp1 and Sp3) that are known to be activators of the expression of type I collagen in human dermal fibroblasts. The work performed by Kypriotou and collaborators demonstrated that c-Krox is a transcriptional activator binding to a region between −112 and −61 bp of the proximal promoter of *COL1A1* and interacting with other *trans*activating factors such as Sp1 and Sp3 [42]–[53]. These factors acted together to *trans*activate the transcription of *COL1A1* in human fibroblasts. Our results revealed that the ASM likely increases the binding activity of Sp1, Sp3, probably CBF and c-Krox. In regards to the WSM, the results are less significant, and a strong increase in the binding activities is not observed, except for the pair of Sp1 and Sp3.

Our results allowed highlighting of an increase in the anabolic pathway of ECM, in particular through stimulation of collagen and GAGs syntheses. However, it is important to note that our results also revealed an increase in the activity and expression of MMP-1 in the presence of shell extracts. The increased expression in the mRNA levels of MMP-1 is correlated to the enhancement of MMP-1 activity after 48 h and 96 h incubation in the presence of ASM and AIM. Our approach allows us to quantify the synthesis of endogenous active MMP-1 without taking into account pro-MMP-1. MMP-1 belongs to the family of collagenases. Its stimulation should lead to a decrease in the amount of ECM components such as collagen. Moreover, our results showed that shell extract stimulated expression in the mRNA levels of p65. This NF kappa B sub-unit is a transcriptional inhibitor of ECM genes like those encoding type I and III collagens and is largely responsible for the decrease in the expression of collagen synthesis occurring during aging of the dermal fibroblasts [53]–[54]. However, our results did not demonstrate such a decrease. Indeed, even if our extracts increased the synthesis of MMP-1, they also stimulated the synthesis of collagens. It appears that the anabolic/catabolic balance could be in favour of collagen synthesis despite MMP-1 activity stimulation. On the other hand, our extracts increased the mRNA levels of TIMP-1. Although we did not evaluate TIMP-1 at the protein level, this is another finding in favour of an increase in the anabolic pathway. Indeed, TIMP-1 is the natural inhibitor of MMP-1, which thereby reduces collagen degradation. Thus, it is not impossible that an increase in the synthesis and activity of MMP-1 may have a beneficial role, as this enzyme is necessary for tissue repair. Indeed, in a skin wound, following the steps of inflammation, tissue repair through the formation of granulation tissue and tissue remodelling occurs via the action of MMPs to maintain a balance in ECM synthesis. In the literature, studies have also demonstrated a concomitant increase in type I collagen, MMP-1 and TIMP-1 in the presence of shellfish extracts. Thus, our results are in agreement with the work performed by Torita and collaborators. These authors demonstrated that human fibroblasts (TIG-103 cell line) cultured and incubated for 96 h in the presence of WSM (450 µg/ml) from *Patinopecten yessoensis* increased the steady-state mRNA levels for type I collagen, MMP-1 and TIMP-1 [30]. Similarly, it has been demonstrated that mouse fibroblasts incubated in the presence of an extract of nacre from *Hyriopsis cumingii Lea* enhanced their synthesis of type I collagen, MMP-2 and -9 and TIMP-1 [55].

Finally, we studied the migratory behaviour of fibroblasts in the presence of shell extracts. The migration and proliferation of fibroblasts represent key steps in the initial phases of skin repair, when dermal integrity is damaged. Fibroblasts will migrate to the wounded site and allow the filling of the wound in synthesising a new ECM. The results concerning the migration of fibroblasts failed to highlight the effects of shell extracts from *Pecten maximus* on cell migration speed. A slight increase in filling the wound could be observed in the presence of WSM but was not significant after 48 h of incubation. In addition, immunostaining of ECM and cell elements involved in fibroblasts migration has been studied during the filling of *in vitro* wounds. The data failed to demonstrate a modification of the location or distribution of actin, vinculin, fibronectin and integrin β1. To our knowledge, few works have studied the effect of shell extracts on the migration of mammalian cells. Lee and coll. [48] demonstrated that a WSM extract from the pearl oyster *Pteria martensii* did not affect the migration of murine NIH3T3 fibroblast after 48 h of exposure. Our results are in accordance with the study of these authors concerning the effects of mollusc shell extracts on mammalian cells motility.

## Conclusions

This study demonstrated that shell extracts, particularly ASM, has effects on the synthesis of ECM components and matrix remodelling. Potential applications could, as a consequence, emerge, particularly in the context of anti-aging strategies. Skin aging is marked by a decrease in the synthesis, among which, collagen. In such a situation, a matrix turnover is beneficial. Moreover, this ECM turnover can also be an advantage in skin repair and scarring. Wound repair is characterised in part by the proliferation and migration of fibroblasts to the injury site and by the synthesis of matrix components such as type I and III collagens. Although our results have not demonstrated effects of shell extracts on cell migration and proliferation, the ECM component synthesis increased in their presence, and this leads to potential opportunities in skin repair systems.

At present, it is not yet possible to identify what molecules present in the shell extracts are biologically active on vertebrate cells. Mollusc shells include a heterogeneous group of soluble proteins, glycoproteins, hydrophobic proteins, chitin and lipids [3], [6], [56], [57]. The acid-soluble protein fraction contains hydrophilic residues, whereas the acid-insoluble fraction rather contains hydrophobic proteins, rich in glycine and alanine. In addition, although many macromolecules are present in the shells, recent studies have also revealed the presence of many low molecular weight molecules: for example, Bédouet and collaborators have identified more than one hundred molecules of low molecular weight in the nacre of *Pinctada margaritifera* [58]. The authors speculate that a number of these molecules correspond to signalling molecules that can be recognised by cell membrane receptors, and subsequently trigger an increase of their metabolic activities. Among these molecules, “cytokine-like” peptides are suspected, but their presence has not been demonstrated. Such molecules may be present in the shell organic matrix of *Pecten maximus*, which would explain the diversity of responses in the biological activities highlighted when examining human dermal fibroblasts.
